# Closing the Gap between Knowledge and Clinical Application: Challenges for Genomic Translation

**DOI:** 10.1371/journal.pgen.1004978

**Published:** 2015-02-26

**Authors:** Wylie Burke, Diane M. Korngiebel

**Affiliations:** 1 Department of Bioethics and Humanities, University of Washington, Seattle, Washington, United States of America; 2 Department of Bioinformatics and Medical Education, University of Washington, Seattle, Washington, United States of America; Stanford University, UNITED STATES

## Abstract

Despite early predictions and rapid progress in research, the introduction of personal genomics into clinical practice has been slow. Several factors contribute to this translational gap between knowledge and clinical application. The evidence available to support genetic test use is often limited, and implementation of new testing programs can be challenging. In addition, the heterogeneity of genomic risk information points to the need for strategies to select and deliver the information most appropriate for particular clinical needs. Accomplishing these tasks also requires recognition that some expectations for personal genomics are unrealistic, notably expectations concerning the clinical utility of genomic risk assessment for common complex diseases. Efforts are needed to improve the body of evidence addressing clinical outcomes for genomics, apply implementation science to personal genomics, and develop realistic goals for genomic risk assessment. In addition, translational research should emphasize the broader benefits of genomic knowledge, including applications of genomic research that provide clinical benefit outside the context of personal genomic risk.

## Introduction

Despite early predictions [1,2], genomic research has not (yet) created a new, more personalized medical care. Many reasons have been offered for this gap between expectations and reality. Some emphasize the evidence deficit: few genetic tests have been demonstrated to improve health outcomes [3,4]. Others point to the slow process of translation, calling for clinician education and decision support to expedite uptake of personal genomics [5,6]. Still others question the proposition that genomics will revolutionize medical care, arguing instead that expectations for personal genomics are inflated [7]. In this paper we explore these explanations and suggest that each offers insights for addressing the gap between genomic knowledge and clinical application.

## Evidence

Many genetic tests have been marketed with scant evidence of clinical value. For example, a guidelines group evaluating *CYP450* testing to inform use of selective serotonin reuptake inhibitors (SSRIs) for depression found no evidence that testing assisted decisions about drug use or improved patient outcomes [8]. Further, *CYP450* genotypes were not clearly correlated with drug levels in people using SSRIs [8]. Clinicians are unlikely to embrace practice change when the evidence for benefit is so uncertain or incomplete.

But how much evidence is enough? The few tests that have moved rapidly into clinical practice suggest that evidence requirements vary. For example, clinical testing for *BRCA* mutations began within a few years of gene discovery, based on strong evidence of clinical validity—that is, evidence for a significant association between mutations in the *BRCA1* and *BRCA2* genes and risk of breast and ovarian cancer [9]—but without evidence of improved health outcomes after testing [10]. The likely explanation for this rapid translation is that clinicians valued a test that could identify which members of high-risk families had inherited the cancer risk. In this instance, clinical validity was sufficient to provide a test with a clear clinical purpose: to guide screening and prevention measures already in use for women at high risk [10].

Gene expression profiling of breast tumors [11] offers a more contested example. Gene expression assays can be used to identify women at low risk of recurrence, who might safely avoid adjuvant chemotherapy, and clinical studies document changes in chemotherapy recommendations with testing [12]. However, there are differences of opinion among expert groups about the evidence. Some consider the retrospective data establishing the clinical validity of gene expression profiling sufficient, while others argue that prospective clinical trials are still needed [13,14]. In fact, randomized controlled trials (RCTs) have played a pivotal role in the uptake of some genetic tests: an RCT demonstrating benefit was key to wide adoption of pharmacogenetic testing for the drug abacavir [15,16]. Conversely, recent RCTs with partially conflicting results have failed to resolve the debate about the value of pharmacogenetic testing for warfarin therapy [17–19].

The issue of adequate evidence is likely to become even more controversial as whole genome approaches are adopted. For example, success in the development of targeted therapies for some tumor mutations has led to increasing use of tumor genome analysis in oncology [20]. Yet tumors are often genetically heterogeneous and develop new genetic changes over time [20]; therefore, assessing appropriate uses and outcomes of this testing approach may require innovative analytic approaches.

These examples indicate that the evidence needed to justify clinical use of a new genetic test varies. For tests that meet a defined clinical need, the evidence requirements are likely to be obvious, and often may not involve RCTs, as *BRCA* testing illustrates. Where the purpose of testing is less clear, or the results difficult to interpret, the evidence requirements are likely to be more stringent, and experts may disagree on the evidence threshold. Expediting genomic translation therefore requires two efforts related to evidence: more clinical research focused on health outcomes, and consensus development concerning acceptable evidence thresholds for different uses of genomic information [21]. Evidence requirements will be particularly important—and challenging to define—as genomics moves from tests of individual genes to whole exome and whole genome testing.

## Implementation Science

Although lack of evidence explains why some genetic tests are slow to enter clinical practice, it cannot explain the poor uptake of tests for which there is strong evidence of benefit. Documented barriers to appropriate genetic testing include lack of genetics knowledge among point-of-care physicians [22] and a dearth of useful (and useable) clinical decision support. In addition, patients may be concerned about the cost of testing and follow-up or have difficulty understanding complex testing protocols. Patient follow-up may be particularly challenging among socioeconomically disadvantaged populations [23]. As these barriers suggest, there is no single “correct” approach to implementation issues, because health systems vary in staffing, location, and capacity of the different clinical specialties involved. In particular, genetics resources (genetic counselors, medical geneticists, specialists with genetics expertise) vary and may dictate differing assignment of responsibilities in different health systems.

A case in point is universal tumor screening for Lynch Syndrome (LS) among patients with colorectal cancer. LS is a condition that confers a high lifetime risk of colorectal cancer and accounts for 2%–4% of colorectal cancers in the US [24]. The Evaluation of Genomic Applications in Practice and Prevention (EGAPP) program recommends screening for LS in individuals newly diagnosed with colorectal cancer to identify patients and family members at risk who will benefit from targeted screening and follow-up [25]. There are many approaches to LS screening, involving choices about the initial screening test, the application of family history criteria at different stages of screening, and the methods used to reach family members when a proband is diagnosed with LS. Successful screening therefore requires local planning to define the preferred screening approach, followed by systematic procedures for implementation of each step of the screening process [26,27]. Given this complexity, it is not surprising that universal LS screening is far from common, with significant variability in screening procedures and low rates of follow-up of patients and family members [23,27,28].

Implementation science, which focuses on identifying and overcoming barriers associated with deploying and tailoring new interventions, offers a means to address the gap between testing capability and practice. The Consolidated Framework for Implementation Research (CFIR), developed by health services researchers at the Veterans Administration, can be a particularly useful framework because it provides a model that can inform both the initial implementation approach and the subsequent analysis that identifies the barriers and facilitators of success that can be used to guide improvements [29]. As summarized in Table 1, CFIR recognizes several domains critical for successful deployment of a new medical service. The application of this framework to LS screening (Table 1) identifies many factors in implementation, and points to specific actions that institutions could take to improve uptake. For example, institutions need to be mindful of local capabilities and the need to coordinate LS screening across the different specialties of oncology, gastroenterology, and primary care. Some suggest the Electronic Medical Record as a way to standardize guideline adherence. However, not all organizations will have the information technology capabilities necessary for successful implementation [30,31]. Institutions may also need to consider the genetics knowledge of the clinicians ordering the tests and the commitment of institution administrators to implementation of a new standard of care. LS screening is likely to be launched successfully only if these issues are taken into account as screening procedures are adapted to local circumstances.

**Table 1:** Consolidated Framework for Implementation Research (CFIR) domains and Lynch Syndrome screening implementation.

Main CFIR Domain	Potential Applications for Lynch Syndrome (LS) screening
Intervention characteristics (adaptability and complexity)	• Clinical decision support (CDS) for heritable colorectal cancer/LS screening, including potential computerized CDS
Outer setting (external policies and incentives/disincentives)	• Federal and state policies • Role and influence of accountable care organizations • Professional practice guidelines • Payer coverage of testing • External incentives (e.g., rewards for better patient outcomes)
Inner setting (structures and climate)	• If computerized CDS, Electronic Medical Record (EMR) software and informatics support, including training for users • Local institutional policy • Organizational climate • Communication patterns and willingness to collaborate across specialties
Characteristics of individuals (knowledge and beliefs about the intervention and role within the organization)	• Identification of key stakeholders across disciplines • Administrator buy-in and leadership • If an EMR-linked CDS, informatics buy-in and leadership • Pathologist engagement • Genetics knowledge of tool users/ordering physicians • Availability and proximity of personnel with genetics expertise • Patient input and needs, including advice on follow-through to improve prevention and family uptake outcomes
Process (planning and executing the intervention)	• Support for planning, testing, evaluation, and iterative improvements

Planning, buy-in, and execution will take different forms for different applications of genomic medicine, but the systematic approaches suggested by the CFIR framework will remain relevant. With the move from programs based on single gene conditions such as LS to genome sequencing approaches, a wide range of implementation challenges will need to be considered, including efficient identification and referral of patients, informed consent procedures, laboratory quality measures, and the scope of secondary findings, unrelated to the clinical problem for which testing was done, to be assessed and delivered. To address these challenges, investments in implementation science will be an important priority for genomics.

## Setting Expectations

As efforts to strengthen the evidence base and pursue implementation science are undertaken, there is a concurrent need to define realistic expectations for personal genomics. The health information incorporated in the human genome is complex and heterogeneous; its value varies according to both the nature of the information and the circumstances of the patient. Information about being a carrier for an X-linked or autosomal recessive disease, for example, is primarily of value for people of reproductive age, and only then if they choose to use such information in reproductive decision making. Similarly, cancer risk information may be highly valuable to a young adult but of little interest to an elderly patient with end stage heart disease. This heterogeneity points to a central challenge for genomic medicine: the need to define the genomic information that is useful in a particular clinical context.

For example, pharmacogenetic testing can improve the safety of abacavir treatment for HIV-AIDS, and thiopurine treatment for acute lymphoblastic leukemia [15,32]. In both cases, pharmacogenetic tests are relevant only in particular clinical circumstances and serve to identify the minority of patients who are at risk for severe adverse reactions, so that an alternative drug or dosage can be used. This information has high clinical utility and points to a future in which pharmacogenomic testing will improve the safety and efficacy of medication use. However, conventional drug choices work for most people; few need individualized adjustment. Similarly, the early benefits of whole genome sequencing have related to gene discovery for rare disorders [33,34] and improved diagnosis of individuals with rare phenotypes whose problems have eluded conventional work-up [35].

Although personal genomics is touted as the means to move from one-size-fits-all to a more individualized approach to health care [20,36], these examples, as well as LS and *BRCA* testing, suggest a different interpretation: genomic risk assessment identifies the minority of outliers who require a modification in treatment or prevention efforts. These successes of genomic medicine underscore, paradoxically, that one size often does fit most, if not all.

In other words, the benefits of genomic risk assessment are important, but have little to do with the health care needs of most people, most of the time. Universal recommendations for vaccinations, Pap testing, and blood pressure evaluation still apply in the era of genomics, and all of us will benefit from well-balanced diets, regular exercise, and avoidance of cigarettes, no matter what genetic predispositions we have. When individual adjustments to care are needed, they most often relate to comorbidities or social circumstances [37]—for example, individualizing an exercise program for a person who uses a wheelchair or adjusting Pap screening recommendations based on HPV status.

As a corollary, genomic risk prediction is likely to be least effective in addressing the population health burdens that matter most—those deriving from common complex diseases such as diabetes, heart disease, stroke, and cancer. Genetics contributes to risk for all these conditions. Rare outliers have high risk due to inheritance of highly penetrant mutations such as those causing LS. More commonly, genetics is only a modest contributor to risk.

For example, variation at over 40 loci is associated with likelihood of developing type 2 diabetes, but a few lifestyle factors account for the majority of risk [38–40]. A recent study evaluating diabetes risk in more than 25,000 individuals illustrates the key role of lifestyle. In this study, a genetic risk score had substantially less effect on absolute risk than obesity: among normal weight individuals, 10-year risk of type 2 diabetes ranged from 0.25% to 0.89% across genetic score quartiles, while for obese individuals the risk ranged from 4.22% to 7.99% [38].

The same general observation applies to virtually all other common diseases: although genetic variation contributes to differences in individual risk, lifestyle and other social determinants of health play a dominant role in health outcomes [41]. Variance in genetic risk for common complex diseases is modest compared to the effect of social factors. Thus, although the genomic risk profile of each individual is unique, most people’s genetic susceptibilities fall within a limited range, from a little below to a little above average [42]. This point is particularly important in considering health disparities. For most people, in the words of Thomas Frieden, head of the Centers for Disease Control and Prevention, “your longevity and health are more determined by your ZIP code than they are by your genetic code” [43]. While the heritability of many diseases is only partially defined [20,39], there is little reason to think that a more complete description of genetic contributors will change this fundamental reality [44].

Further, identifying risk for common diseases is generally not difficult: a variety of metrics, including weight, blood pressure, and biomarkers such as cholesterol and hemoglobin A1c, are available for this purpose. Assisting people to make behavioral changes to reduce their health risks is more difficult. Yet it has been estimated that hundreds of thousands of premature deaths could be avoided by reducing smoking, improving diet, and increasing activity levels [41]. As a corollary, public policies related to availability of healthy food, safe places to exercise, access to smoking cessation programs, and research on lifestyle modification are likely to be better long-term investments for improving public health than providing genetic screening for addressing common disease risks.

To be sure, genomic risk assessment is still of value, and opportunities to improve health outcomes through genomic screening are likely to increase over time. As the LS example illustrates, clinical translation of such discoveries will require both evidence for improved outcomes from screening and systematic efforts to implement screening programs. In addition to single gene conditions like LS, further research may point to ways in which genomic risk profiles can be used to identify outliers with high cumulative risk for complex diseases [42]. As this knowledge accumulates, there will be increasing justification for genome-scale screening to ensure that high-risk individuals are offered appropriate targeted care. In some instances, genomic risk profiling could provide benefits in cases not limited to high-risk conditions like LS: for example, a genomic risk profile for cancer could conceivably outperform family history as a means to identify individuals with moderately increased risk who are candidates for early breast, colorectal, or prostate cancer screening [45]. The potential harms of screening [46] and the many nongenetic contributors to risk suggest, however, that this type of genomic profiling will require robust evidence of benefit [47].

It remains the case that genomic discovery related to the major disease burdens of the population will yield many variants of small effect. For most such findings, there is no translational gap in personalized medicine to overcome, because the information lacks clinical value. Instead, there will be an increasing need for analytic, technical, and clinical strategies that pull from the genome the information that can improve health care, while avoiding the introduction of large amounts of poorly predictive and distracting health information into the medical record.

## Conclusion: Moving Beyond Personal Genomes

Although most gene variants associated with common complex diseases will be poorly predictive and lack clinical utility for individual health care, they nevertheless represent highly valuable research findings. Every gene causally associated with disease is a marker for a biological pathway, potentially revealing unexpected mechanisms of disease, connections between different pathological processes, and interactions between biological pathways and environmental risk factors. Promising examples are proliferating at a rapid rate. For instance, genome wide association studies (GWAS) have clarified the importance of autophagy in the pathogenesis of autoimmune disease, identified new loci associated with disease, and pointed to shared pathways between inflammatory bowel disease susceptibility and host responses to mycobacterial infection [48]. In age-related macular degeneration, GWAS played a pivotal role in clarifying the importance of the complement system in pathophysiology of the disease [49], leading to new insights for therapy. Studies of the genetics of type 2 diabetes similarly provide insights about the relationship between this disorder and cardiovascular disease [50], the role of immune factors [51], and diverse contributors to insulin resistance [52]. These examples underscore the power of genome-scale research methods that do not rely on prior biological hypotheses for gene discovery, and point to the increasing potential for progress as researchers move from GWAS to whole genome studies [53].

Genomics is a source of an expanding set of molecular tools for other avenues of health research as well [20]. For example, there is a growing body of research demonstrating the powerful effect of epigenetic changes in gene expression as a source of disease risk [54]. These studies may provide new insights into social determinants of health and could conceivably inform social policies related to issues such as maternal and early childhood nutrition or other environmental exposures relevant to health.

Genomic research can also improve care by defining the genotypes of other organisms. In a recent widely publicized case, an infectious agent was identified and treated through the use of DNA-based diagnostics [55], pointing to the growing use of genomics in pathogen identification [56]. This use of genomics is an early indicator of an expanding role for the genomics of pathogenic and symbiotic organisms, with applications including the assessment (and potential amelioration) of the microbiome [57] and the use of genomics in vaccine development [56].

These examples provide only a glimpse of the potential benefits of genomic health research. However, they offer an important insight about closing the gap between genomic knowledge and clinical care: the task is not solely, or even primarily, one of learning how to use individual genomes in clinical care [58]. A person’s genotype undoubtedly offers useful information in some clinical circumstances, but much of the benefit will come from leveraging genomic knowledge to develop a more precise understanding of molecular physiology. Such efforts point to a different way in which the translational gap may be closed: by developing prevention and therapeutic strategies that provide benefit outside the context of genetic risk.
